# Voltage Gated Ion Channel Function: Gating, Conduction, and the Role of Water and Protons

**DOI:** 10.3390/ijms13021680

**Published:** 2012-02-06

**Authors:** Alisher M. Kariev, Michael E. Green

**Affiliations:** Department of Chemistry, City College of the City University of New York, 160 Convent Avenue, New York, NY 10031, USA; E-Mail: alisher@sci.ccny.cuny.edu

**Keywords:** potassium ion channels, gating, conduction, protons, water

## Abstract

Ion channels, which are found in every biological cell, regulate the concentration of electrolytes, and are responsible for multiple biological functions, including in particular the propagation of nerve impulses. The channels with the latter function are gated (opened) by a voltage signal, which allows Na^+^ into the cell and K^+^ out. These channels have several positively charged amino acids on a transmembrane domain of their voltage sensor, and it is generally considered, based primarily on two lines of experimental evidence, that these charges move with respect to the membrane to open the channel. At least three forms of motion, with greatly differing extents and mechanisms of motion, have been proposed. There is a “gating current”, a capacitative current preceding the channel opening, that corresponds to several charges (for one class of channel typically 12–13) crossing the membrane field, which may not require protein physically crossing a large fraction of the membrane. The coupling to the opening of the channel would in these models depend on the motion. The conduction itself is usually assumed to require the “gate” of the channel to be pulled apart to allow ions to enter as a section of the protein partially crosses the membrane, and a selectivity filter at the opposite end of the channel determines the ion which is allowed to pass through. We will here primarily consider K^+^ channels, although Na^+^ channels are similar. We propose that the mechanism of gating differs from that which is generally accepted, in that the positively charged residues need not move (there may be some motion, but not as gating current). Instead, protons may constitute the gating current, causing the gate to open; opening consists of only increasing the diameter at the gate from approximately 6 Å to approximately 12 Å. We propose in addition that the gate oscillates rather than simply opens, and the ion experiences a barrier to its motion across the channel that is tuned by the water present within the channel. Our own quantum calculations as well as numerous experiments of others are interpreted in terms of this hypothesis. It is also shown that the evidence that supports the motion of the sensor as the gating current can also be consistent with the hypothesis we present.

## 1. Introduction

**Figure 1:**
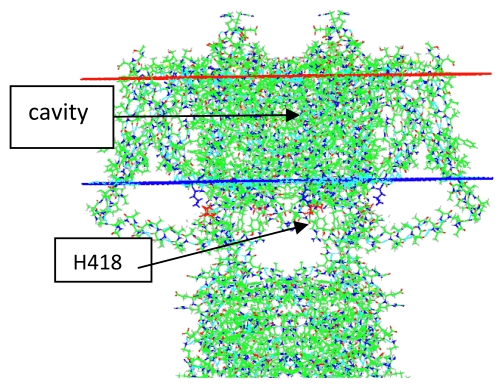
General view of the channel structure, taken from the 2A79 structure. Two domains out of four are shown and the membrane position is shown as the two horizontal lines. The gate is below the inner membrane boundary (blue), and the cavity runs from approximately the inner membrane about 2/3 of the way to the upper membrane boundary. (red). One important amino acid, referred to below, and one section, are labeled.

Ion channels are ubiquitous in biological cells. In bacteria, they regulate the concentration of Na^+^, K^+^, and some other ions. The channels we are most concerned with are in eukaryotes, and are, among other things, responsible for the nerve impulse. However, we will see that the differences between a bacterial channel and a eukaryotic channel are surprisingly small. These channels have been studied for decades, and the basic information regarding their electrophysiology, and other information that was available up to 2001, has been summarized by Hille [1]. In this review we will be concerned primarily with voltage gated K^+^ channels and will also consider the large amount of information on the related bacterial KcsA K^+^ channel, which is gated by a drop in pH, rather than voltage. We will look at the function as well as the structure of the channels, and will consider the unorthodox possibility that the gating current may be created by the motion of protons through the voltage sensing domain (VSD) of the voltage gated channels. The general structure of the channel is shown in Figure 1.

There has been general agreement that the S4 segment of the VSD, which contains arginines or lysines every third residue over a stretch of 4 to 8 residues must move to create a *gating current*, which precedes the ionic current that begins with the opening of the channel. The conventional view is that the mechanical motion of the S4 transmembrane (TM) segment creates the gating current by moving the positive charges on the arginines of that segment across (most of) the transmembrane field. There are several versions of the conventional view, each proposing a different form of the motion of the key S4 segment. There is dispute as to whether all or part of the S3 segment also moves, and general agreement that the S1 and S2 segments do not move with respect to the membrane. However, there are also interactions between the channel and the phospholipids of the membrane. There is no doubt that protons are capable of moving through the VSD, but fairly general implicit agreement that this must not happen during normal gating. The agreement is implicit, in that the question is hardly ever raised. The existence of a pore in the VSD of the channel (the omega pore), together with the fact that protons would necessarily move in response to a change in potential, means that the *absence* of a proton current would require some explanation. In addition, it is known that mutating the end arginines allows a proton current through the membrane. There is also a voltage gated proton channel with strong homology to the VSD. It is conceivable that all the p*K* values of residues lining the pore (S4 arginines and the negatively charged residues to which they are salt bridged) are so far from the relevant pH range that the state of ionization of all residues cannot be changed even with the voltage applied in the simulations. However, we will see that there is much more reason to believe that the p*K* shifts into a range that allows change in the ionization state of even the arginines of the VSD, and the explanation cannot be simply the p*K* values. The huge amount of data has led to multiple reviews of the channel gating mechanism, as well as conductance and selectivity, since Hille’s book; a few, fairly recent examples include reviews by Bezanilla, by Roux, and Boiteaux *et al.* [2–4].

There is a great deal of data that shows that there are relations among the various parts of the channel, and a large part of the channel protein is “below” (that is, intracellular to) the membrane surface. (We will use the convention that “up” is extracellular, “down” intracellular.) This includes a gondola-like segment, called T1, for tetramerization domain. It has been shown that this domain, although seemingly remote from the gate, is also involved in gating. There is also a relation between the lower end of the channel and the selectivity filter, which plays a primary role in selecting for potassium, and is near the upper end of the channel. In addition to opening and closing, the channel *inactivates* by two mechanisms: a fast inactivation that is accounted for by an intracellular group, and slow inactivation by a mechanism that appears to connect the gate to the lower end of the selectivity filter. Between the gate and the selectivity filter there is a cavity which contains a number of water molecules; the exact number is in some dispute, and, as we shall see, probably varies as the ions pass during the channel opening. Since the VSD must be linked to the gate, essentially all parts of the channel must work together in order to have a functional channel. There is also evidence of cooperativity among the four TM domains [5].

This review compares a hypothesis concerning the functioning of the various sections of the channel to several “conventional” models, and the evidence supporting these models. Conventional models have in common that they have channel activation dependent on one of several versions of a conformational change in one transmembrane segment, S4, of the VSD. Each of these has been extensively reviewed over the past several years from the points of view of the various forms of conventional models. Therefore a detailed discussion of these is not given here. The new model, which does not require any particular movement of that TM domain is then described, and experiments and calculations that we consider easier to interpret on the new model introduced. One part of the hypothesis, concerning the role of proton transport, is not new; it has been previously discussed several times, and reviewed [6]. However, other sections, including the oscillating gate, are new, and there is new evidence that must be discussed in the context of the model. The final section includes, briefly, some suggestions for possible experiments that might distinguish this model from conventional models. There are two primary questions we must investigate: what is the evidence bearing on the nature of the charges responsible for gating a voltage gated ion channel, and how is the progress of the ion through the channel modulated by interactions at the gate? Interactions with the T1 intracellular domain and a barrier to the progress of the ion at the selectivity filter must also be considered. This leads to the second part of the hypothesis, in which the gate oscillates, forming a short lived complex with the ion as it transits the gate. This in turn leads to a model, albeit qualitative, of slow inactivation. At least one major aspect of the gating picture is omitted; there is no detailed discussion of the kinetic substates along the gating path that are revealed by electrophysiological studies. It is clearly possible for protons to move stepwise, but the exact physical counterparts of the kinetic steps are not yet a part of the model. These experiments do not clearly distinguish the new hypothesis from the conventional models.

We summarize the main points of the new model, including gating current and activation gate:

- Gating current consists of protons. In the present form of the model, there are three H^+^ per domain, leading to a gating current of 12 charges. In K_v_1.2, measured gating current is approximately 13 charges. There are multiple ways to get an additional approximately ¼ charge per domain, including partial charge transfer that may be associated with hydrogen bonds, and dipole rotation.
- The path of the protons is along the aqueous pathway that gives rise to the omega current in VSD. This parallels the S4 transmembrane segment. It is similar to the pathway for the protons in the H_v_1 proton channel, save that there is no selectivity filter for protons analogous to the D112 [7] in the H_v_1 channel.
- Key residues that accept the protons at the intracellular end include H418 (S6), E142 (T1), and E136 (T1). The importance of H418 will be discussed below, as it has already been reported to be necessary for channel function [8].
- We have not defined the open state locations for the protons yet. They would have to stop below the outermost arginine, as mutating that to histidine allows proton current through the VSD (which also confirms that protons are capable of moving through the VSD, and that they are near the arginines). It is also possible that lipids help define the extracellular proton reservoir, although the evidence for that is at this point weak.

### The Channel

**Figure 2:**
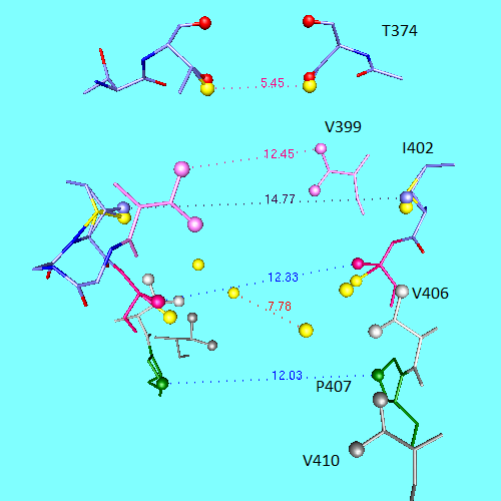
The cavity of the KcsA channel compared to that of the K_v_1.2 eukaryotic K^+^ channel. The near identity of the upper section of the cavity, in spite of KcsA being a closed, K_v_1.2 an open, configuration, is remarkable, especially when it is added to the huge evolutionary spread between prokaryotic and eukaryotic channels. The lower section shows the difference of conformation between open and closed, with the diameter of the opening approximately 12 Ǻ instead of 6 Ǻ, showing how small the difference is between open and closed structures. The yellow spheres are carbon or oxygen atoms from KcsA, while the amino acids are from the 3Lut structure of K_v_1.2, as labeled on the figure. The atoms marked by a distance of 7.78 Ǻ near the gate (bottom) of the figure are the carbons of KcsA T107 to which the −OH of the threonines are attached, and the 5.45 Ǻ distance at the top corresponds to the hydroxyl oxygens of T75 of KcsA, and to the equivalent atoms of T374 of K_v_1.2. The labeled residues are from K_v_1.2.

Figure 1 shows the structure of a voltage gated K_v_ channel, K_v_1.2, or *Shaker*, in the open state, as determined by X-ray crystallography; the figure is based on the pdb structure 2A79 [9]; as shown it is pdb structure 3Lut, derived from 2A79 by normal mode analysis, and showing hydrogens, by Chen *et al.* [10]. The figure shows two of the four VSDs at the sides of the central pore region looking at the side of the channel. A figure of the bacterial KcsA channel would show essentially the same pore region, but no VSD. The other major difference is that the KcsA structure is in the closed configuration. Figure 2 shows a comparison of the central cavity region of the two channels. What is remarkable is that the upper section of the cavity, as well as the selectivity filter, are nearly identical, in spite of the fact that the channels are separated by hundreds of millions of years of evolutionary history. The upper sections of the cavity, and the whole of the selectivity filter, have very similar dimensions. The eukaryotic channel has an extra isoleucine, in place of a phenylalanine, making it somewhat more hydrophobic. The fact that one is open and the other closed is reflected in the lower section at the gate; the difference between open and closed conformations is about 2–3 Å in radius.

This figure is already enough to tell us that the structure is strongly conserved and presumably cannot accept very much in the way of substitutions without losing its function. The sequence TVGYG in the selectivity filter is conserved through almost all K^+^ channels, with an occasional conservative substitution for the valine. At the gate, opening the channel requires that there be about a 2–3 Å motion of one residue on the S6 transmembrane (TM) segment which lines the pore, and smaller motions of the neighboring residues. The conventional models produce this motion by having the S4–S5 linker pull back about this distance, or, in some versions, much more. It is this conventional view that we wish to compare to the consequences of proton motion on the gating section of the channel, and related differences in the way the channel functions. The KcsA channel, lacking any VSD, gates directly with a drop in pH, while in our model the voltage gated channels are gated by the rearrangements of protons in response to the depolarization of the channel. In conventional models, it is the mechanical motion of part of the VSD that is responsible for the gating.

We first examine the conventional models, and the evidence from which they are derived. We shall then define our alternative, and the evidence that is compatible with this model. In the third section, we shall extend our model to show that it can be compatible with a wider range of channel functions, including inactivation. We shall consider the questions that remain unsettled, including a possible role for lipids. Finally we suggest two classes of experiments that might distinguish the present hypothesis from conventional models.

## 2. Conventional Models

“Conventional models”, as just defined, are those that derive the gating current of voltage gated channels from the physical motion of the S4 TM segment of the VSD. To open the channel, the S4 with its positive charges moves in an extracellular direction upon depolarization of the membrane, pulling the S4–S5 linker, thus separating the intracellular ends of S5 and S6. The wider space makes room for the ions to pass from the intracellular fluid to the channel pore. The S4 has arginines every third residue, except in one, and sometimes two, locations, which have lysine in place of arginine, generally near the intracellular end. These are all residues that would be expected to be positively charged at physiological pH, as they would be in solution. Even for the arginines, though, it is possible that in the protein the charge state varies. We consider three standard classes of models, including some modifications: (i) the “paddle”[11]; (ii) the “helical screw” (which also has undergone a number of changes); (iii) a model providing alternating access from the intra and extracellular sides, when the channel is closed, and then open. This resembles a transporter [12]. All of these depend primarily on three lines of evidence: access from solution to mutants of the S4 (and sometimes other) TM segments from the VSD; the most important mutations are R→C, followed by access to reaction with methanethiosulfonate (MTS) reagents with the thiol of the cysteine. For this reaction (thiosulfonate bonding to a thiol to form a –S–S– bond) to occur, the cys must ionize into the form R–S^−^; reaction with R-SH itself is over eight orders of magnitude slower [13]. This is evidence of a minimum p*K* shift in the appropriate direction of about 1.5 p*K* units from the value in solution. The primary finding is that when the channel is open, reaction, detected by the loss of channel function, occurs when the mutation is on an outer arginine, while when closed, reaction occurs on cysteine residues replacing inner arginines. This is interpreted to mean that the S4 has moved from the inner (closed) position, where the inner positions are accessible, to the outer position, where the extracellular residues are accessible. The 2A79 (& 3Lut) structure shows S4 having its last two arginines near the outer membrane leaflet, as would be appropriate for these models in the open state. The second line of evidence is based on the same mutations, but considers the change in gating charge on neutralization of the S4 charges [14]. Third are the fluorescence resonance energy transfer experiments (FRET), which tend to show fairly small motion. The basic experiments in these classes were done relatively early, and were reviewed by Bezanilla [15] The three classes of conventional models are not compatible with each other, when considered in detail.

### 2.1. The Paddle

Here, not only S4, but part of S3, designated S3b, must move up 15 Å or more to open the channel [16]. The reagents used to label the channel are much larger than the MTS reagents used in any other experiments. The reagent attached to S4 is biotin, using a chain of varying length; a protein external reagent, streptavidin, is used to link to the biotin. The point of using such a large external reagent is to ensure it remains at the membrane surface. Presumably then the distances measured are not altered by the external reagent entering the membrane itself, changing the distances determined with respect to the membrane surface. Assuming all the linkages are stretched as postulated, this would make a well-defined system. The salt bridges between S4 and at least part of S3 do not need to break during this motion.

### 2.2. The Helical Screw

There are a number of versions of helical screw models. Horn has provided a good relatively early summary of a model, related to the helical screw [17] that examines this mechanism based in large part on cys substitution data. An NMR study also supports a version of this model [18]. All of these postulate that the S4 twists as it moves up, with the arginines exchanging salt bridge partners on the S2 and S3 segments as S4 moves. Therefore this is quite different from the paddle. It is less clear how the energetics work out as the arginine twists away from the negative charges on S2 and S3 (see comment below); a number of proposals have been advanced, making varying assumptions as to the local water, dielectric constant, and transformation of part of the segment from α-helix to 3_10_ helix. Each claims to have an energetically reasonable path, but we will not try to evaluate these here; it is enough to note that paths have been proposed, with approximate energy estimates that seem plausible, given the assumptions on which they are based. Evidence for this version of a conventional model includes double cys substitutions with cross-linking at different points depending on the state of the channel [19]. However, among other problems with this experiment, the time allowed for the cross-linking is much longer than for gating, allowing for extensive fluctuations, which may also be larger in mutant channels. This model requires a pore within which to twist, compatible with the finding of the “omega current” which allows ions and substances up to roughly the size of a guanidinium ion to pass through the VSD, using the pore. The existence of an aqueous pore within the VSD [20,21] can be considered established. It was discovered by Isacoff and coworkers, and has been found as well by others [18]. The models proposed by Tombola [20], with Isacoff and coworkers, are included in this category. The pore may also conduct protons, a point that will be important to us. Here we note that the basic idea of having the S4 segment thread its way through the VSD still is based on access measurements. The fact that a guanidinium is the largest ion that can pass the omega pore has been taken as a further indication that the pore is the right size for S4 to move up; it presumably would not be appropriate for the helical screw, which would require a larger pore, but might work for a conventional model in which S4 moved straight up. However, that would produce difficulties, including the barrier to breaking multiple salt bridges.

### 2.3. A Comment on Both the Paddle and the Helical Screw

The negative charges on the S2 and S3 TM segments of the VSD [14,22], referred to above, are required for channel function. If these move, their charges would have to be included in the gating charges, whether adding (if they move down), or subtracting, if they move up. In principle, they could move in a region of low field, so make little contribution, but as they are salt bridged to the key arginines, it is hard to see how this is possible. In the helical screw, they do not move; the screw turns the arginines so as to exchange partners with successive negative charges. However, S3 access is state dependent, in the *same* direction as S4 [23]. If the motion of the charges is in the same direction as S4, there should be fewer gating charges, something that requires some difficult interpretation to understand. In the paddle model, the S3 segment splits; it is not entirely clear how to make this compatible with the Nguyen and Horn results [23].

### 2.4. The Alternating Access Model, and Its Descendants

Bezanilla and coworkers made a critically important observation: there appears to be an extremely (on the scale of biological systems) high field, of at least 10^8^ V·m^−1^, in a very localized region of the VSD. This was determined by placing a fluorescent moiety on each of the positions of the arginines of S4. One of these shows the existence of the high field [24]. This field allows a smaller motion of charges to produce the full gating current, and can be combined with fluorescence resonance energy transfer (FRET) as a measure of motion. (Most of the results come from using a lanthanide ion as one of the components of the system, and the method is therefore designated LRET.) This group and some others find, based on FRET results, a much more limited movement, especially vertical movement [25]. A later paper from the same laboratory suggests a slightly larger movement [26], but still appreciably less than what would be expected from the access evidence described above. Also, the fluorescence data often show less vertical movement even if the total movement is not greatly less than that suggested by access measurement; this is still incompatible with the access results. The rest of the movement is “horizontal”, parallel to the plane of the membrane. Earlier results reported even less movement [27]. An independent study by Ahern and Horn, using tethered charges, also supports a high field/short motion mechanism [28]. A study of deletions in the S3–S4 extracellular linker also suggests that large movements of the S4 segment are unnecessary for gating [29]. The high field allows a small vertical movement because the charges still cross the majority of the membrane voltage drop. A field of 10^8^ V·m^−1^ across 4 Å represents a 40 mV drop, equal to 10^7^ V·m^−1^ across 40 Å, which would be the case for a uniform field across the entire hydrophobic section of the membrane. Since the high field reported by this group of workers is ≥10^8^ V·m^−1^, the relatively short vertical movement is still compatible with the observed gating current. Even though a relatively short pull on the S4–S5 linker can open the channel, this is still a “conventional model” as we have defined it, because the gating current comes from the motion of the arginines of S4. The access results are attributed to opening and closing of clefts on the extracellular surface, open in the open state of the channel, and on the intracellular surface, open in the closed state of the channel. This alternating access model resembles transporters. We will account for the access to MTS reagents by a somewhat similar model.

We have summarized the conventional models very briefly. There is vastly more literature than cited here, especially on the matter of access to cysteines substituted along S4. It is clear that there are state dependent differences in access to the mutant cysteines, but we argue that this does not imply that the gating current must consist of simply the motion of S4, whether a large motion (paddle, helical screw) or a small motion across a high field. The access difference in the last model can be attributed to opening clefts at the external and internal sides of the VSD. The interpretation of changes in gating current with charge mutations is also not so obvious, as some experiments are more consistent with a proton path interruption than with simple carrying of charge.

## 3. The New Model

### 3.1. Gating Current Derived from Proton Transfer (Proton Current)

Just as there is no question that access to cysteine mutants in the arginine positions is state dependent, there is no question that proton transport along the VSD from one side of the membrane to the other is possible. The evidence includes the fact that mutation of arginines at either end of S4 to histidine produces a proton current through the entire VSD [30,31]. It is also the case that if the amino acids between the arginines are mutated to histidine, there is no such current. If the histidines were protonated, and the protons carried physically across a barrier, then the proton current would be found for these mutants as well. The absence of this current is evidence that it is the protons themselves that are moving. There is also a channel that bears a very strong resemblance to a VSD, which is itself a voltage gated proton channel, discovered by DeCoursey and coworkers [32,33] designated H_v_1 [34,35]. There have been proposals for the proton path in the latter case [36,37]. At this point we do not want to discuss the exact path, which also depends on the mutations that make the H_v_1 not exactly like the VSD. However, it is clear that a transfer of protons across the membrane is possible. In the conventional models this is ignored; instead, these models assume, generally implicitly, that the protons must not move at all if they do not move entirely across the membrane. If there were any proton motion, then it would be necessary to explain how the protons fail to contribute to the measured gating current. In the conventional models, there is no gating current contribution from protons, and no comment on this point. One further type of evidence has not been discussed: there have been extensive calculations on channels, the majority molecular dynamics (MD), but also some quantum calculations. One such quantum calculation shows that a proton can move from an arginine that is ionized to one that is not, when the space between is bridged by water molecules, and the distance between arginines is as in S4 [38,39]. While no surprise, given the experimental evidence, this confirms that the hypothesis is reasonable on this point.

### 3.2. The Oscillating Gate

There is another new part of the model: we predict that the activation gate oscillates as each ion passes through the gate. We discuss this in detail below. An ion in the cavity would repel an ion approaching the gate, and the ion at the center of the cavity would be unable to reach the selectivity filter without a boost from an external field appreciably greater than what is provided by the applied field. Therefore there must be a complex for the ion at the gate, to hold it against the central ion, and to thus push the central ion into the selectivity filter over the barrier there. We show below that the energy required for the complexes is reasonable, although full calculations remain to be done.

### 3.3. There Are Parts of the New Model Taken from Earlier Work by Others

There are parts of the model that are not new, that have already been proposed by others. This includes interactions of the VSD with the lipids of the membrane, and the interaction of the activation gate and slow inactivation. We will discuss the evidence bearing on these points as well.

## 4. Evidence Supporting the New Parts of the Model

These are experiments that we believe can be more easily interpreted on the present model: protons as gating current.

### 4.1. A Key Mutation Cuts Gating Current

If a charge is mutated *between* S4 arginines, adding a new charged residue in place of an uncharged residue, the gating current is further limited [40], *regardless of the sign* of the amino acid added in the mutation. The simplest (not the only) interpretation would seem to be that the proton current is blocked by the added charge, if positive, or trapped, if the charge is negative. On the conventional models this should be considered surprising, in that the sign of the charge is not relevant. If the charge is positive, and the S4 moving, it seems that more charge should move across the field, while negative charge would produce less; this seems almost unavoidable in the paddle and helical screw models. Even in the alternating access model it seems at least puzzling. However, experimentally, gating current is found to be less either way; while not definitive evidence for protons being the gating current, it is easier to understand the result if gating current is carried by protons. This experiment in particular contradicts the interpretation of the lower gating current in R→C mutations as implying that the arginines physically carry the current.

### 4.2. The “Piquito”

Another experiment that is easier to understand if the gating current is carried by protons is the initial small extremely fast rise observed by Stefani and Bezanilla, which they have called the “piquito”. An attempt to fit this into the standard model was made by Sigg and these authors [41–43]. However, the explanation assumed a complex energy landscape that was not clearly defined. It also requires fluctuations that are difficult to understand in the sense that solving the accompanying Fokker-Planck equation appears to lead to fluctuations, while the measurement appears to be of a deterministic phenomenon. It is not appropriate to critique this explanation here; the observation itself is clearly very important, and it is the observation from which we begin. Proton tunneling would exactly mimic the observations, without invoking any complex explanations. For quantum calculations that show how such a step might occur, albeit in a model system, see Yin and Green [44]. Since an initial proton move could initiate a cascade of protons, this is just what would be needed on the model being introduced here. Depolarization of the membrane would allow matching of energy levels for proton transfer, possibly by tunneling. Transfer of a proton would allow a cascade of protons to follow, as the initial motion was followed by replacing the proton that moved, followed by one or perhaps two successive transfers. The fluctuations of the initial motion would be a consequence of a threshold of matching energy levels. There would be a sigmoidal distribution of open probability, which could be indistinguishable from the Boltzmann model [45,46].

### 4.3. A Switch at the Gate

**Figure 3:**
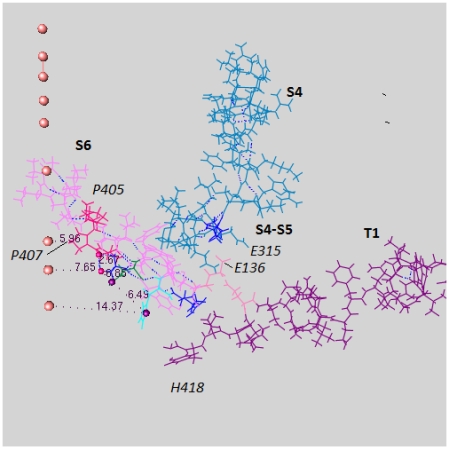
The “switch” that opens the gate with surrounding regions, which are shown to orient the view, from the 3Lut structure: Note that there are several salt bridges that may be affected by the addition of ions, particularly H^+^. Segments are labeled in boldface, individual residues in italics. Of residues referred to in the text, P407, E136, and H418 are labeled. The orange spheres are on the pore axis, and the distances shown are in the plane normal to the axis, measured from carbon atoms in the residues, except for the P407 distance, measured from nitrogen in the ring. Note that this makes the open diameter less than 12 Ǻ. Blue atoms are carbon, red, oxygen, purple, nitrogen, green, hydrogen;

**Figure 4:**
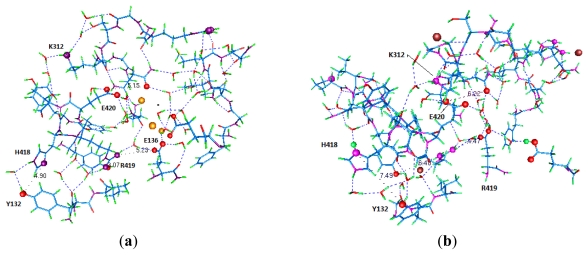
Figure 4a,b shows the same section of the channel, a part of the section shown in Figure 3 (see labels on the amino acids). Figure 4a shows the open state (it uses 3 positive ions, K^+^, for an approximation to the potential in the open state, and in fact there was very little change from the X-ray structure upon optimization); Figure 4b has been optimized with 2 H^+^ + 3 Cl^−^ added (the 3 Cl^−^ make the internal potential negative). The model would then make this a representation of the closed state. There is movement of certain residues with respect to the S4–S5 linker, in Figure 4b, with respect to either the X-ray or 3 K^+^ structures (which are similar). Several residues have been labeled explicitly here. Two residues have taken the two protons in this calculation, H418 and E142, and we expect the third proton to go to E136 when it is included in the calculation. Colors: as in Figure 3, plus potassium, large gold spheres (Figure 4a), chloride, large brown spheres (Figure 4b); oxygen and nitrogen atoms shown as large spheres are involved in transferring protons, with a proposed path for protons indicated by hydrogen bonds (from gOpenMol). Certain interatomic distances are indicated to show the difference between the conformations with protons and without. There is a small but significant conformational change apparent between Figure 4a and Figure 4b; the distance from the nitrogen in H418 to the oxygen in Y132 has expanded from 4.90 to 7.49 Ǻ, with the ring flipped, essentially enough to account for the closing of the gate. Because of broken salt bridges, K312 and R419 have also moved. Optimizations used Gaussian09 [50] at HF/6-31G** level. Figure 4a has 524 atoms, Figure 4b has 615 atoms (both, including water). Outer backbone atoms were frozen to provide a framework replacing the forces exerted on this region by the remainder of the protein.

Next we consider the intracellular end of the channel. Figure 3 shows the complex of amino acids from several sections at this end of the channel. These include residues from the T1 moiety which hangs below the channel in the intracellular space, as well as part of the *C*-terminal of S6 and part of the S4–S5 linker. In the conventional models, it is hard to see how the T1 moiety participates in gating at all, but it does [47–49]. Adding protons to this region can cause a rearrangement of highly conserved residues, closing the channel. The X-ray structure shows the open state, and Figure 4a,b shows how the difference between protonated and unprotonated forms could account for the opening and closing of the gate of the channel. Figures 3 and 4 show that a plausible fit to the new model can be achieved. Based on a recently completed calculation, the possible intracellular locations for protons in the closed state are shown in Figure 4.

### 4.4. D_2_O Shows a Role for Solvent in the Final Step of the Gating Process

When D_2_O is used as the solvent, gating is slowed [51–53]; specifically the last stage in gating is slowed [54]. There is no obvious way that this can be understood in models in which water is not, in part at least, critical to the process of gating. This does indicate that the opening of the activation gate requires the participation of the solvent; in none of the conventional models does water play a role at the activation gate. All require as their final step a mechanical pull on the pore S5 segment by the S4–S5 linker, and the role of water is therefore difficult to understand in these models.

### 4.5. Pressure: Osmotic vs. Hydrostatic

There is further indication that the water plays a role, albeit possibly indirectly, from osmotic pressure experiments. There appears from several lines of evidence to be a final step that opens the channel. The D_2_O slowing referred to above is consistent with such a final gating step involving the water. Hydrostatic pressure shows an apparent activation volume of approximately 60 Å^3^ [55–57] suggesting two water molecules are forced into the channel as it opens, if we take water at bulk density. However, Zimmerberg *et al.*, using osmotic stress [58], estimated 40–50 water molecules are involved with gating; clearly this is far more than could be associated with the cavity. Following this work, an osmotic stress study by Starkus *et al.* [59] also concluded that increasing osmolarity slowed conduction. The latter study considered unilateral osmotic stress and non-equilibrium effects, with complex results, including three steps for gating. It appears that intracellular stress slowed the first step in opening the channel, while there is a large effect on the final step in opening in response to extracellular osmoticants. Again, the amount of water is too large to be accommodated by the pore. Therefore it must be associated with the VSD. Access of water is not seen particularly as a property of S4, but of the VSD as a whole. The osmotic studies may also be consistent with a smaller quantity of water leaving the channel pore. If the osmotic pressure results suggest a much larger number of molecules than can be associated with the cavity, or with conduction, what is it that they do measure? The difference in access of external reagents (e.g., MTS reagents) upon channel opening suggests that the water may be entering the VSD. We have discussed access changes for the arginines of S4, and also for S3. What is being observed is likely increased access to the VSD segments lining the omega current pore from solution (in this the discussion resembles the transporter model) as the association with lipid weakens (see the discussion below of the role of lipids). This would allow the water to enter the VSD from the intracellular end on channel opening, and would account for the large magnitude of the osmotic effect reported by Zimmerberg *et al.* [58] The possibility that this also involves lipid molecules is not considered in that discussion, but it is not excluded; indeed, it seems probable. One of these studies [60] used a toxin, and hypothesized that the role of the toxin was to alter the membrane-channel interaction. We agree that that is reasonable, and hypothesize further that the toxin alters the ability of water to enter the region where the channel and the membrane interact. Butterwick and MacKinnon more recently found that lipid interacted with the VSD of the KvAP channel [61].

Conti *et al.* consider *hydrostatic* pressure results to correspond to an appreciable rearrangement during gating [57]. These are measurements on Na^+^ channels, but again the similarities to K^+^ channels ought to be good enough to take the results to be essentially the same. These measurements show a much smaller effect than the osmotic pressure experiments, corresponding to roughly two molecules of water for the slowing of the rise of the ionic current; the effect on gating kinetics gives an activation volume of approximately one molecule of water. This would be appropriate for the number of water molecules that might enter the pore cavity with each ion, and should affect the conductivity more than the gating. (such a small Δ*V* (activation) may be difficult to reconcile with large gating movements.) In hydrostatic pressure experiments the free energy of the water rises, while with increasing osmotic pressure the free energy of water decreases. The two effects can therefore relate to different parts of the channel cycle. The apparent activation volume of gating derived from the effect of hydrostatic pressure may actually relate to the first entrance of an ion into the pore, which would be consistent with the slowing of the rise of the ionic current.

### 4.6. Non-Linear Response to Large Gating Voltages

There is one other set of experiments that is included in this discussion for the sake of completeness. It has been generally ignored over the quarter century since it was published, and has not been considered in any of the previous models. It has been neither repeated, nor refuted. However, if correct, this result reinforces the interpretation of the “piquito” (experiment 2 above) that we have given. Fohlmeister and Adelman found non-linearities in the gating current of an ensemble of sodium channels [62,63]. These are consistent with a threshold in gating, which would be appropriate for the “piquito”. It has been shown that the threshold can produce essentially the same sigmoidal gating current-potential curve as the standard Boltzmann assumption, as well as the non-linearities that are observed [45,46]. This also comes with a prediction of stochastic resonance, but on this there is no pertinent literature, as far as we are aware. It does suggest a testable prediction. However, it must be acknowledged that this work was not done on a single channel system, and probably much more needs to be done before the result can be fully interpreted.

This concludes the list of experiments relating to gating current that appear to be easier to interpret on the new model; some are particularly difficult to understand on the conventional models, including the piquito, the D_2_O results, and the role of T1. Others support the new model, but are not so difficult to understand as these three on the standard models. While the arguments are not conclusive, they are strong enough to make the new model at least worthy of consideration.

Next, we have to consider the nature of the gate and its relation to conductivity. In the standard model, gate opening merely creates a hole through which the ion passes on its way to the interior of the pore, then on to the selectivity filter. However, the conductance is a function of intracellular K^+^ concentration, unlike a normal conductor, which does not have conductance proportional to the external concentration of charge carrier. Therefore, the channel acts as something other than a normal conductor.

## 5. The Key Evidence Supporting the Oscillating Gate

### 5.1. The Gate and the Intracellular Solution

**Figure 5:**
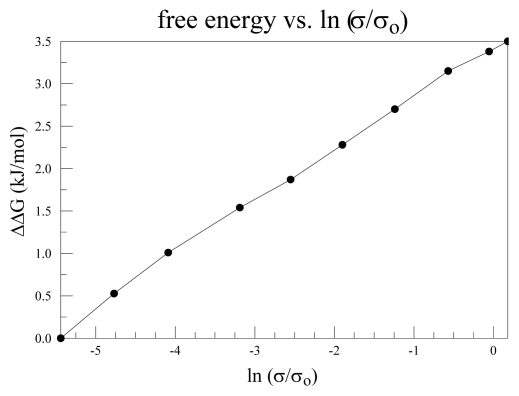
The conductance-intracellular K^+^ concentration curve: This is plotted as log [K^+^] *vs.* log *σ*, in both cases relative to the lowest concentration, from Le Masurier *et al.* [64]. Since log [K^+^] is linearly proportional to free energy, this is a Δ*G*-log *σ* plot, in effect. It indicates that increasing the free energy of the potassium ion is consistent with the ion having to surmount a barrier in its path, and our calculations suggest that this barrier is in the cavity.

**Figure A2:**
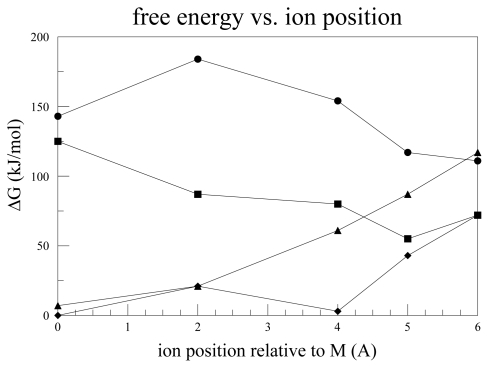
The free energy *vs.* position curves for 12, 14, 16, and 18 oxygen molecules. There is a large barrier to getting to the center of the cavity with 12 or 14 molecules, but much less with 16 or 18 water molecules in the cavity. It is possible that the ion pushes out one or two water molecules as it moves up, or that the water content is affected by the presence of an ion at the gate, as suggested in Figure 6. The free energy calculated here does not include a second ion; the interaction of a second ion with the ion calculated here would raise the energy by a decreasing amount as the water moves up, for any number of water molecules. The calculation is corrected for the free energy of the appropriate number of water molecules, compared to bulk solution. The energy of the system was calculated, as in Figure A1, with the ion in each of the following positions: M (middle of cavity), M + 2, M + 4, M + 5, and M + 6, where the last is the lowest position of the selectivity filter:numbers of water molecules: circles, 12; squares, 14; triangles, 16; diamonds, 18.

In Figure 2 we saw that the change in the channel pore conformation was not large between open and closed states. The upper part of the cavity hardly moved. The lower part of the cavity moves only a few Angstroms. The conductance of the channel has been shown by LeMasurier *et al.* to depend on the intracellular concentration of K^+^ [64], at least for KcsA. While not a voltage gated channel, the pore region is essentially identical to that of the K_v_ channels. Conductance of a normal conductor does not depend on the concentration of the charge carriers outside the conductor itself. However, (Figure 5) we see that it is possible to understand this if we plot log [K^+^] *vs.* log (conductance) from the Le Masurier *et al.* paper. The log [K^+^] dependence means a linear dependence on Δ*G*(K_es_^+^) with K_es_^+^ in intracellular solution, of the log(conductance). The most obvious way to get such a dependence is to have a barrier to the progress of the ion through the cavity, with the ΔΔ*G* = Δ*G*(K_es_^+^) – Δ*G*(K_sf_^+^) (with free energy measured from the energy of the ion in the infinitely dilute solution, each term on the right hand side is a Δ*G*), K_sf_^+^ = ion at entrance to the selectivity filter. We combine that with a calculation of the ion’s progress in the cavity region. This has not been previously published, and will therefore be described briefly in the appendix. The essential result is that there is a barrier to the progress of the ion in its path in the pore, and the height of the barrier is dependent on the number of water molecules in the cavity. This number may vary as the ion moves into the cavity from the intracellular solution. The calculations presented in the appendix (Figure A2) determine the energy of the ion as it moves up the cavity toward the selectivity filter. The calculations are done separately for each number of water molecules.

The most serious error in absolute value of the calculated energy is probably the limitation on the number of atoms that can be included; this should not vary greatly with the number of water molecules. However, the general point that there is an energy minimum near the center of the cavity with a climb to the selectivity filter would be hard to change in future work. This has an interesting consequence, to which we now turn.

### 5.2. Minimum at the Gate, Required for the Knock-on Mechanism

If there is a free-energy minimum in the center of the cavity (not necessarily in the geometric center, but fairly close—the X-Ray structure shows an ion in this position), then it is necessary to have the ion in this position moved along by some sort of potential. For, say, a 50 mV potential applied across the membrane, the energy is lowered by about 2 k_B_T, or about 5 kJ for the entire membrane, too small for what is needed to cross the barrier into the selectivity filter. When added to the terms covering other barriers, as we are about to see, the external field produces an increase in current, so the measured I–V curve can be understood. The conductivity for potentials in this range is roughly ohmic, so considering the external voltage as the means of overcoming the internal barrier is unlikely to be correct. If one considers two ions, one at the gate, the other at the cavity midpoint, approximately 6 Å distant, the coulombic energy of repulsion is 4 × 10^−19^/*ɛ* J, where *ɛ* is the effective dielectric constant. This *ɛ* is likely to be larger than the *ɛ* at the gate; there is more water, and it is freer to move. While the system is more polarizable than the interior of a protein, it should be much less so than bulk water. If *ɛ* = 10, the energy is 4 × 10^−20^ J, or about 10 k_B_T, approximately 25 kJ. If *ɛ* is as large as 30, the energy is still over 10 kJ, more than the energy of one charge with 50 mV across the membrane (even if the entire drop were in this region, which would mean something else would have to boost the ion through the remainder of the channel, and no such thing is known). At the gate, there is less water, a higher field, and tighter hydrogen bonding. This is the reason we consider the dielectric constant to be in the 10 to 30 range here, compared with perhaps 4 at the gate. The ion approaching the gate will be repelled by an energy greater than that provided by the voltage across the membrane, so would be expected to fail to reach the gate, or if it does so, to progress past the gate. The classical “knock-on” mechanism would have the incoming ion push the central ion forward toward the selectivity filter. However, an equal and opposite force pushes the incoming ion back out of the gate. The obvious way to solve this problem is to have a free energy minimum for the ion at the gate, so that the ion is complexed in place long enough to force the central ion upward. In order to have this minimum, the protein must do the complexing; even if the complex is too weak to isolate, with a binding energy of only, say, 25 kJ, it should suffice to hold against the central ion. A wide open gate would not work, as an incoming ion would effectively be in bulk solution, and would be pushed back. From Figure 2 we saw that the X-ray open structure expands from about a 6 Å carbon-carbon distance across the opening to about 12 Å. This is a C atom distance, so the space for the ion is smaller. Possibly an electrostatic minimum could form at the gate from the dipoles of the neighboring protein, as suggested by Chung *et al.* [65]. However, if the well is of constant depth, the depth would have to be greater than that at the center of the vestibule. If the well is constant, then it is hard to see how the ion in that well could move, or an ion that approached the gate from the intracellular side not be repelled. All that happens in that case is that the position at which the “knock-back” effect takes place moves down slightly. The number of ions that can diffuse to the channel on the intracellular side, depending on the detailed assumptions concerning the area of the channel that is taken as the region the ion must reach, would have to be at least the observed current; with no barrier this is possible. A 13 kJ barrier would reduce the number by exp (−13/2.5) ≈ 6 × 10^−3^, enough to make it impossible to have an adequate current. We therefore seek a mechanism that does not have this problem. We consider instead the consequences of an oscillating gate.

### 5.3. Oscillation of the Gate

**Figure 6:**
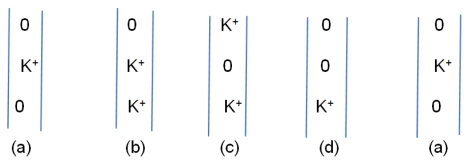
A possible oscillation sequence for the ions as they progress in the channel: the three positions are: bottom, gate; center; cavity center; top, S4 (lowest) position of the selectivity filter. States (b) and (d) are expected to be short-lived compared to (a) and (c).

The oscillating gate we consider has two states: closed/complexed, with an ion in the gate, and open/separated. In the latter case an approaching ion can be pulled in, with the gate plus ion switching to the closed/complexed conformation. The next step is to push the center ion up, leaving a vacancy at the center; after this, the complexed ion at the gate can move to the center, allowing the gate to reopen for the next ion. Thus there is a cycle of states, with one example suggested in Figure 6. The existence of minima requires either that the rate of random ion movements be great enough to allow passage of ions, with the external voltage sufficient to drive the current, or the oscillatory mechanism described here. We can consider the energetics of the external field: there is only about 5–10 kJ of energy available, compared to the several times that, that would be minimally necessary. Therefore, the oscillatory hypothesis appears to be a better fit to what is known. The oscillation that is required is only about 2 to 3 Å from each domain, approximately the distance moved by H418, shown in the difference between Figure 4a,b. The gate should be able to reflect this motion. The second requirement is for a complex of the right strength. A calculation of the strength of the complex, whether by threonine (T107) in KcsA or proline (P407 in K_v_1.2) in K_v_ channels, is in progress. However, ab initio calculations of a single prolylglycyl dipeptide with K^+^ show a gas phase complex [66]; with 4 prolines (4 domains), such a complex should easily be strong enough, even in solution. The strength is about consistent with an order of magnitude estimate. (The upper limit on the strength of the potential well would be determined by the minimum at the cavity center; a too-deep minimum at the gate would also prevent conduction).

The order of magnitude estimate goes as follows:

$\Delta E\approx (1/4\pi {ɛ}_{\text{o}})\text{q}\mu (1/{r_{\text{c}}}^{2}-1/{r_{\text{o}}}^{2})/ɛ$, where *r*_c_ is the distance between dipole μ and the charge q in the complexed state, *r*_o_ in the open state. Taking *μ* = 2 D, *r*_c_ = 3 Å, *r*_o_ = 6 Å, *ɛ* = 4, we get Δ*E* ≈ 12 kJ. For the complete complex, with four domains, assuming no interference of fields of the dipoles, this makes close to 50 kJ. This is probably more than is necessary, but is very much in the right neighborhood. Of course, we have chosen a dielectric constant and dipole strength that, while plausible, are not supported by direct evidence. In addition, a macroscopic calculation is not entirely correct either. With these caveats, we can say that the proposed complex has a strength that is not unreasonable.

The complete quantum calculation that is needed is just beginning. One reason we need to do the complete calculation is that the correlations among the hydrating molecules at the ion would almost certainly make the total energy less than four times the single interaction; with 12 kJ for a single domain, perhaps 30–40 kJ would be more likely for 4 domains.

The ion path through the cavity has several minima, of which the three most significant are at the gate (so far lacking a quantum calculation), at the center of the cavity, and the S4 position of the selectivity filter (the latter two supported by the calculations summarized in part (A) of this appendix). This suggests looking at several possible sequences of states. We offer just that given in Figure 6 above. Although this omits possible changes in number of water molecules during the cycle, it provides a reasonable sequence of states for an oscillating gate.

There is a further implication of the oscillating gate. Either the gate, or a nearby part of the protein, must be able to contract around the ion. Then if an ion that makes a strong complex is used, it should stick in the gate. This experiment has already been done, with Cd^2+^, with a cysteine mutation at the gate [67,68]. It is possible to form the expected complex. This is enough to show that there is a state in which the gate can be bridged by a single ion.

Having come this far, there are some implications of the model that must be accounted for to make the model self-consistent. For one thing, if the protons are to proceed from one side of the membrane to the other, they have to have a place to stop moving at the external end when open, and the internal end when closed.

### 5.4. The Proton Reservoirs

While proton transport through the VSD seems to be well established, there will still be no such contribution to the gating current if there is not a way for the protons to be accepted at either end of the S4 segment. The protons that travel from the intracellular to the extracellular end of the VSD when the channel opens, and in reverse when it closes, must have a location at which they can be accepted at each end. Otherwise, the proton current would continue through into the solution. However, such a current is only found when the end arginines are mutated to histidine. Evidently the protons are blocked by the charges on the end arginines. Also, the gating current is thus limited. Membrane lipids may also play a role. These reservoirs are more than “parking spots” for protons, as the protons at the inner end must actually close the channel, just as a proton opens the KcsA channel. Amino acids H418, E136, and E142 appear to be the center of the intracellular (closed state) reservoir (again, see Figure 4b). There is no mechanical pull on the gate coming from the S4–S5 linker in this model, although that linker does play a critical role in transmitting the protons. If it is put together correctly, the KcsA channel can be attached to a VSD and made into a voltage gated channel [69]. If the linker in a voltage gated channel is disrupted, there can be gating current without opening the channel [70]. The structure at the intracellular end is conserved. In any model, including ours, this is necessary. The extracellular reservoir has not yet been defined as well, although it must be below the outermost arginine (R1).

### 5.5. A Role for T1

We have discussed the interaction of the T1 moiety with the gate; the relevant section of a single domain of K_v_1.2 was shown in Figure 3 and Figure 4. These figures include several water molecules, showing a series of linked hydrogen bonds; these bonds show a path that the protons would be expected to take to reach the gate, and move the key histidine (H418). This is based on a calculation (Kariev and Green, unpublished) of this region; the calculation has been done under three conditions: starting from the X-ray structure with no added ions, with three added K^+^, and with three added Cl^−^ plus two H^+^. We can see the motion of the H418 in Figure 4b. Since the computation cannot, being static, show an oscillation, and the ions in the computation are forced to find a single minimum, there is still more to compute to obtain dynamics. However, it is clear from this much that the freeing of the gating end of S4–S5 and the role of T1 can be understood if extra H^+^ are present, even if we neutralize the overall charge (here, Cl^−^ does this, in addition to adjusting the potential).

## 6. Lipids

The channel must interact with the surrounding lipids. Schmidt and MacKinnon [71] showed that negatively charged lipids are needed for channel functioning. KcsA interacts with lipid, according to MD evidence [72], as do other channels [73]. Since these MD results work with essentially electrostatic forces, they ignore quantum effects. Hydrophobic coupling has been suggested for a channel and lipid [74]. Toxins have been used to demonstrate coupling [75]. Other proposals for a role for lipids have been based on lipid exposure of the VSD; for example a role for externally applied polyunsaturated fatty acids (PUFAs), which would have to reach the VSD by way of the membrane, has been found by Borjesson and Elinder, albeit with some channel specificity [76]. The same group found that certain PUFAs, but not saturated fatty acids, could shift the I–V curves of the Shaker channel in a negative direction, and that there was a pH dependence of this shift [77]. The fact that saturated fatty acids failed to have an effect was interpreted to mean that they lacked access to the relevant region of the VSD. The fact that there is pH dependence as well is consistent with the new model, although it could also be an electrostatic effect as the authors proposed. Mechanosensitivity of voltage gated channels has been shown [78]. Furthermore, Schmidt and MacKinnon also showed that the mechanical state of the membrane affects voltage gating [60]. Where the question of the lipid charge has been considered, the lipids appear to be negatively charged. This makes sense if the lipid makes a complex with arginine, as suggested based on quantum calculations [79], and supported further by MD simulations [80]. There is evidence that not all arginines at the extracellular end are ionized. If the arginine is not ionized, and there are negatively charged lipids, then the reservoir needed for protons in the “up”, or open, state, must exist. While there are places for protons, the hypothesis that there are locations for specifically three to four protons remains to be proven.

## 7. Slow Inactivation

Slow inactivation is connected to the intracellular activation gate [81–86]. However, slow inactivation involves the selectivity filter at the extracellular end of the channel, the opposite end of the channel from the activation gate. There have been multiple interpretations. Cuello *et al.* [85] presented evidence that the coupling involves the F103 residue in the middle of the KcsA cavity lining, and comparable residues in other K^+^ channels. They attributed this to a mechanical coupling involving hydrogen bonds from the activation to the inactivation gate. However, the evidence included a simulation involving water molecules. The water would be strongly affected by the way in which the wall is perturbed by the substitution of a smaller residue for the phenylalanine in the mid-cavity wall. Both the space available for the water and the arrangement of the hydrogen bonds would be altered. Therefore, although we will focus on water in the following discussion, we do not necessarily disagree with the Cuello *et al.* interpretation; rather, we add to it, and consider the alteration of hydrogen bonds involving water, and the solvation of the ion, as of primary interest from our point of view. The importance of F103 and corresponding residues is consistent with this view, in that the partial rearrangement of the cavity wall would force rearrangement of hydrogen bonds. In fact, if the protein in the cavity wall remained rigid, it would be hard to see how the water would rearrange as it must, which appears to require that the residues in the cavity wall play a role. In our calculation as well, we see that the phenylalanine that Cuello *et al.* identified plays a role, and rotates so as to affect the water.

We have carried out calculations of the water in the KscA cavity with a K^+^ ion in the closed configuration, as well as some more limited calculations of an upper half cavity (hence, no difference in whether it was open or closed). We used 12, 14, 16 and 18 water molecules, with the latter being approximately the density of bulk water. That assumes an effective radius of 5 Å for an equivalent sphere, hence a volume of about 525 Å^3^, compared with 540 Å^3^ needed for 18 water molecules at bulk density. Of course, the ion occupies about as much space as a water molecule, making 16–17 molecules a more appropriate number and, more important, the ion organizes the water in the vicinity. Therefore, although the density is only approximate, it seems safe enough to cover the range of 12 to 18 water molecules plus one ion. The system has been optimized (using HF/6-31G**) with the ion in the center of the cavity, as well as with the ion fixed 2, 4, 5, and 6 Å above this position. The latter position is the S4 position of the selectivity filter. Calculations, as noted earlier, are discussed in the Appendix. The key finding from the calculation is that there is always a barrier to the ion entering the selectivity filter. The barrier is small with 14 water molecules, but the energy is too high at the center for this configuration to contribute. There is a large barrier to the entrance of the ion to the cavity; if we assume 16 water molecules (12 or 14 lead to very large barriers), it is close to 100 kJ. There is also the possibility, not directly considered in the calculation, that the number of water molecules fluctuates during the cycle of gating and transition of the ion from gate to selectivity filter. The oscillating gate mechanism can allow the water in addition to the ion to flow in and out during the cycle, requiring that the hydrogen bonds break and reform with the passage of each ion. This allows a small probability that the bonds will reform so as to allow a fluctuation in hydrogen bonding to the amino acids anchoring the boundary such that the cycle is interrupted. Because there are multiple possibilities for such a rearrangement, it is likely that different calculations will lead to alternate configurations of the inactivated state. Fluctuations occurring with a frequency of about 1 in 10^6^ to 1 in 10^7^ ions would seemingly give the correct rate, and a probability in this range is plausible. More extensive calculations would be needed to establish this rate. At the moment, we merely note that the experimental evidence concerning slow inactivation is consistent with this mechanism, and the calculations that we have done are most easily understood in these terms; we find it difficult to see a good alternative explanation. Furthermore, the existence of a barrier, combined with the oscillating gate, ties the activation and inactivation gates together.

The oscillating gate is needed to allow the ion to enter the cavity at all; a free energy minimum at a static activation gate still does not prevent the ion from getting trapped there, and repelling following ions, unless it is released by the gate. Since the oscillation required is approximately 2 to 3 Å in radius, this is not a major conformational change, but should lead to a major change in free energy of the complex of the K^+^ ion. In the model presented here, the role of water is central to the conductivity of the ion, and having one to two water molecules that must move into the pore per ion is the best estimate that can be made from the calculations. Static calculations cannot give an exact number, but only an estimate based on free energy. Fluctuations must be allowed for, and thus the number is necessarily approximate. However, the mechanism suggested here is consistent with the experimental data to the accuracy of both the experiments and the calculations. Slowing conduction at higher osmotic stress is qualitatively consistent with this view. Oscillation of the gate does not make any obvious prediction with regard to an osmotic effect.

## 8. Discussion

It is now time to put the pieces together. We have set forth the separate sections of the model: proton transport as gating current, the source and sink for the protons, probable interaction with membrane lipid and the reason for the requirement for interaction with the lipid, together with the relation of the lipid to the cysteine accessibility measurements, the interaction of the activation gate with the T1 moiety, and the reason for the dependence of conductivity on the intracellular concentration of K^+^ in terms of the oscillating gate. We also have a mechanism for the interaction of the activation and inactivation gates by way of the water in the cavity, and the relation of this to the cavity lining protein structure. This last is required at least qualitatively by our results as well. Furthermore, we can account at least qualitatively for the dependence of gating and conduction on osmotic pressure, as well as the gating dependence on D_2_O. Also, the barrier that we find in the quantum calculations can be related to the [K^+^] dependence of conductance.

### 8.1. Proposed Experiments

We consider the channel as a single system that has multiple interlinked parts. The separate experimental results on the VSD, on the pore, on the S4–S5 linker, and on other separate sections, can be given specific interpretations. Here we attempt to fit the parts together to make a fully functioning single entity. One additional purpose of this review is to suggest experiments that could test the ideas presented here, and the experiments that remain to be done are largely combinations of two or more effects, such as osmotic pressure with access. So many individual experiments have been done without agreement on a single model, that it is likely that a new approach is required; it may produce a single version of a standard model, or a model more like that considered here. However, whichever turns out to be correct, so far the evidence points in more than one direction, and clarification is needed.

There are two major differences between what we propose and all the standard models; the gating current and the oscillating activation gate. The main experiments that support the standard models are cys mutants, affecting both access and gating current, and FRET experiments. This suggests the following as possible new experiments that would distinguish the model proposed here from these models.

### 8.2. Osmotic Pressure Combined with Access

One experiment has been suggested above: combine osmotic pressure with access. Standard models would predict no interaction, with the access the same with or without an osmotic pressure difference. However, if water is pulled out of the VSD, then we would expect that access might be more difficult at higher osmolarity, slowing the rate of cys modification. In addition, the number of molecules of water in the osmotic effect would be reduced if another molecule filled part of the space the water would normally occupy. In contrast, if the S4 TM segment were moving to the surface, the osmotic effect on access should be very small or non-existent.

### 8.3. A Possible Means of Detecting an Oscillating Gate

The proposed oscillating gate suggests looking for almost any effect that can be measured independently, and that interacts with the gate at a rate comparable to that at which the ions pass through the gate. While in principle an NMR experiment could show a shift in response to rates of 10^7^ to 10^8^ s^−1^, this comes in the frequency range where NMR has the most difficulty. However, ESR line broadening might also work. In that case the rate should increase with the current, hence with the applied potential, and the line broadening would be affected accordingly. The calculations for this have not been done, but the experiment should be possible with a spin label near the gate.

## 9. Conclusion

A model is proposed for ion channel gating that differs from previous models in that it attributes the gating current to protons, and proposes an oscillating activation gate. A variety of experimental results can be more easily explained in this way. Experiments that have been used to support the conventional models can also be understood in this model, and at least qualitative explanations are given for these. Some new calculations are also offered in support of the proposed model, as well as two proposed experiments.

## Figures and Tables

**Figure A1:**
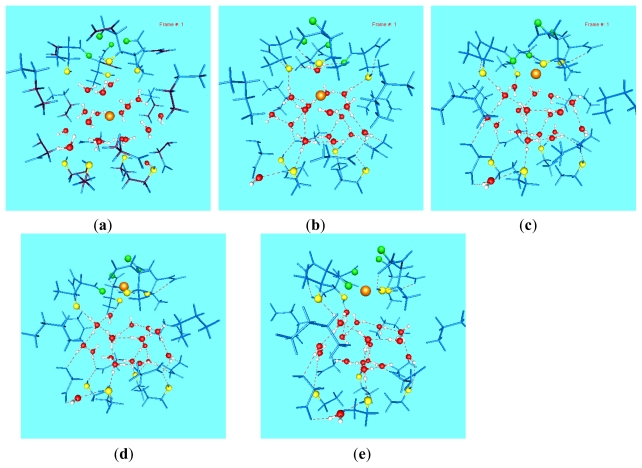
The positions of the ion and the accompanying water molecules and section of channel included in this computation, for the case of 16 water molecules. The ion is the large orange sphere, water oxygens are red, threonine oxygens yellow, and carbonyl green. From the upper left corner clockwise, the position of the ion is M (mid-cavity), M + 2, M + 4, M + 5, M + 6 Å (the latter being the lowest position of the selectivity filter).The figures show the results of optimization at HF/6-31G** level; experience has shown that there is very little difference in geometry when optimization is done using DFT. However, DFT is used for calculating free energy from frequencies with the optimized structures, as used in Figure A2 (B3LYP/6-31+G**). The outer carbons are frozen at the X-ray positions, and all other atoms are optimized.

## Appendix A

The progress of the ion through the cavity can be followed by first optimizing the structure, and then determining the energy at room temperature from a frequency calculation. We have done this for 20 cases of positions of the ion in the cavity of the KcsA (closed) channel. It may seem odd to choose a closed conformation, but if the channel must oscillate, with an ion at the gate while another ion moves from the center of the cavity to the S4 (lowest) position of the selectivity filter, the configuration is correct, except that the lower ion is omitted. Therefore the energy of each conformation represents the contribution of the water and the amino acids to the ion’s progress in the upper half of the cavity. The contribution of the ion in the lower half should be approximately the same for all cases, and the principal effect would be to push the ion forward. Therefore it makes sense to show the calculations. Only one case is shown here, with 16 water molecules plus the ion (Figure A1). In addition, Figure A2 shows the free energy as a function of position for 12, 14, 16, and 18 water molecules, illustrating that the lowest energies may be reached by changing the number of water molecules in the cavity. While this is an incomplete study, it does illustrate how the path of the ion is linked to the water and the gate must be linked to the selectivity filter. Further work is needed to add the details.

## Appendix B

The ion has several minima, of which the three most significant are at the gate (so far lacking a quantum calculation), at the center of the cavity, and the S4 position of the selectivity filter (the latter two supported by the calculations summarized in part (A) of this appendix). This suggests looking at several possible sequences of states. We offered just one here, that shown in Figure 6. Here we suggest a more general notation for such sequences. The number of water molecules may vary during the passage of the ion but this is not included in this notation, although a modification is straightforward. We can postulate that the path of the ion includes states with one or else two ions. Define the notation (*x*, *y*, *z*) as the minima for gate, center cavity, and S4 (selectivity filter), where *x*, *y*, *z* = 0,1, where 0 means unoccupied, 1 means occupied. As a sample sequence, we might start from (0,1,0) (as in the closed X-ray configuration). If there is a suitable minimum at the gate, the second state could be (1,1,0), followed rapidly by (1,0,1), as the ion at the gate pushes (“knocks on”) the ion that started at the center, to the S4 position. If the ion in the S4 position can go downhill to the next position in the selectivity filter, this becomes (1,0,0), and if the center position has lower energy than the gate, this goes to (0,1,0), which returns to the initial state. This leads back to the sequence illustrated in Figure 6. While it should be possible to propose another sequence, and full calculations are needed, this shows that a plausible sequence can be found, and shows how an oscillating gate could function.
